# A Case of Pigmented Bowen Disease on the Finger Mimicking Melanoma Showing a Chaos Pattern on Dermoscopy

**DOI:** 10.5826/dpc.1004a79

**Published:** 2020-10-26

**Authors:** Ryoji Kurita, Yaei Togawa, Keisuke Suehiro, Hiroyuki Matsue

**Affiliations:** 1 Department of Dermatology, Graduate School of Medicine, Chiba University, Japan

**Keywords:** pigmented Bowen’s disease, melanoma, parallel furrow pattern, parallel ridge pattern, dotted vessels

## Case Presentation

**Figure 1:**
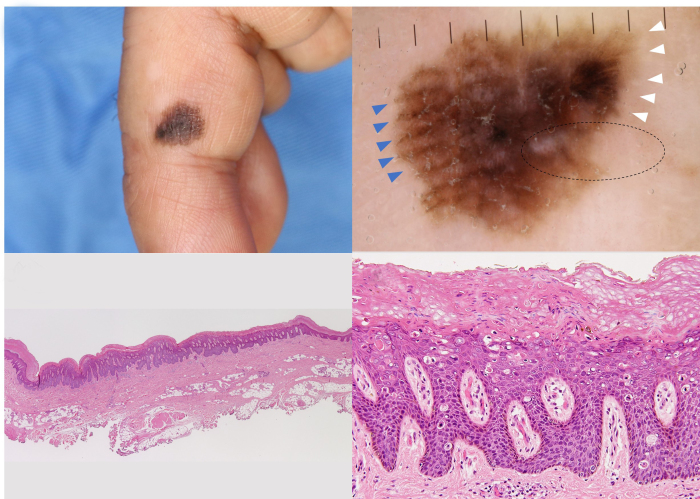
(A) A black macule was seen on the right ring finger, measuring 8 × 6 mm. (B) Dermoscopy shows multicomponent chaos pattern composed of eccentric blackish brown structureless areas from the center to right side of the lesion, some scattered whitish clods, parallel lines on both the furrow and ridge on the left side (blue arrowheads), segmental radial lines from the upper to right rim of the lesion (white arrowheads), and linear arrangement of dotted vessels in the lower right rim of the lesion (black dotted circle). (C) Histopathology revealed hyperkeratosis and acanthosis with elongation and thickening of the rete ridges in the epidermis (H&E stain, lower magnification). (D) In higher magnification (H&E, ×200), atypical epidermal cells with loss of cell polarity and some atypical individual cell dyskeratosis in the epidermis and melanin deposition in the basal layer were seen. HMB45, S-100, and Melan-A stains were negative (data not shown).

A 53-year-old man presented with a 5-year history of a slowly increasing black macule on the palmar side of the ring finger (Figure 1A). Dermoscopic examination showed a “chaos” pattern composed of scaly surface, whitish clods, eccentric blackish brown structureless areas, parallel lines on both furrow and ridge, and peripheral segmental radial lines (Figure 1B). Total excision was performed, and histopathologic features were characteristic of Bowen disease (Figure 1, C and D).

## Teaching Point

Pigmented Bowen disease of the palm and sole is a rare subtype of Bowen disease that mimics melanoma [1,2]. Two previous reports described a multicomponent pattern with regularly distributed dotted vessels and peripheral parallel furrow/lattice-like pattern and/or parallel ridge pattern [1,2]. In our case, we found the following dermoscopic signs suggesting a melanoma diagnosis: atypical parallel pattern along both the furrow and ridges in the left part of the lesion and a more chaotic pattern with segmental radial lines on the right. Other dermoscopic features were instead suggestive of Bowen disease, such as a scaly surface and linear arrangement of dotted vessels.

## References

[1] Cavicchini S, Tourlaki A, Ghislanzoni M, Alberizzi P, Alessi E (2010). Pigmented Bowen disease of the palm: An atypical case diagnosed by dermoscopy. J Am Acad Dermatol.

[2] Nako T, Hoashi T, Mayumi N (2017). Case of pigmented Bowen’s disease on the volar aspect of the finger dermoscopically mimicking melanoma in situ. J Dermatol.

